# Cohort Effects on Tobacco Consumption and Its Genetic and Environmental Variance Among Finnish Adults Born Between 1880 and 1957

**DOI:** 10.1093/ntr/ntae091

**Published:** 2024-04-17

**Authors:** Stephanie Zellers, Hermine H M Maes, Antti Latvala, Jaakko Kaprio

**Affiliations:** Institute for Molecular Medicine Finland, University of Helsinki, Helsinki, Finland; Virginia Institute for Psychiatric and Behavioral Genetics, Department of Human and Molecular Genetics, Psychiatry and Massey Cancer Center, Virginia Commonwealth University, Richmond, VA, USA; Institute of Criminology and Legal Policy, University of Helsinki, Helsinki, Finland; Institute for Molecular Medicine Finland, University of Helsinki, Helsinki, Finland

## Abstract

**Introduction:**

Population research indicates that smoking behaviors in Finland have varied over time by sex and birth cohort. Smoking behaviors are influenced by genes and the environment; like the behaviors themselves, these underlying influences are not necessarily stable over time and may be modifiable by national drug policy.

**Aims and Methods:**

We utilized longitudinal mixed-effects models and causal–common–contingent twin models to evaluate sex and cohort effects on tobacco consumption and the underlying genetic and environmental variance components in a birth cohort sample of same-sex twins born in Finland between 1880 and 1957, assessed in 1975, 1981, 1990, and 2011.

**Results:**

We identified significant main effects of age, sex, and cohort on quantity of cigarette consumption, as well as significant age × cohort and sex × cohort interactions. We also identified sex and cohort effects on the liability to initiate regular smoking and the magnitude of variation underlying quantity of cigarette consumption. That said, heritability and environmental contributions to both traits were not different between the four sex × cohort groups.

**Conclusions:**

Our results indicate sex and cohort effects on the prevalence of smoking and its underlying variation. Our results on changing prevalence mirror existing population-level research in Finnish samples, but we did not identify differences in heritability found in other studies of cohort effects in tobacco use, potentially due to power issues. These results highlight the importance of considering age, cohort, and timing of policy changes when evaluating changes in substance consumption across time.

**Implications:**

This study identifies sex and cohort effects influencing tobacco consumption in a sample of Finnish adult twins born between 1880 and 1957. Our results are in line with other population-level research in Finland and research on cohort effects influencing alcohol use in the same sample. Our results highlight the intertwining effects of age, cohort, sex, and substance policies on substance use.

## Introduction

Tobacco consumption is a leading cause of premature mortality and health care spending, and it is influenced by both genetic and environmental factors.^1–5^ Tobacco consumption and its underlying genetic and environmental influences are not necessarily stable over time. In fact, the prevalence and quantity of tobacco consumption and its underlying variation changes with age.^1,5^ Furthermore, tobacco consumption has changed with birth cohorts and the way that tobacco consumption changes across the lifespan may itself depend on birth cohort.^6–11^

Several well-powered population studies in Finland found a decline in prevalence of smoking with increasingly recent birth cohorts.^6–8^ Similar studies in American twin samples found birth cohort effects on the genetic and environmental influences on tobacco consumption. Specifically, genetic influences on regular smoking were largest in individuals born between 1925–1935 and 1951–1956, whereas they were lowest for those born in the early 1940s or mid-1960s.^10^ These varied hertiabilities across birth cohorts are associated with different mechanisms of gene–environment interplay: social control, social trigger, and social push.^10^

These studies also suggest that cohort effects in tobacco consumption and its underlying variation were linked to changes in tobacco policy, like the Finnish 1976 Tobacco Control Act,^6^ the Finnish 1995 Tobacco Control Act Amendment,^7^ and the American Surgeon General’s Warning of 1964.^10^ Not all tobacco policy changes have been associated with changes in heritability though; tobacco policy changes in the Netherlands that reduced the prevalence of smoking did not change the heritability of smoking across birth cohorts.^12^

These varied findings across countries highlight the importance of the environment to studies of heritability and cohort effects, as there are many reasons that could explain why these studies found different results.^9,10,12^ For example, drug control policies and social attitudes around substance consumption are likely intertwined, and this is the case for both tobacco and alcohol.^10,13^ A study on alcohol consumption in Finland reported birth cohort effects on the variation underlying alcohol consumption, potentially due to cultural changes around the value of abstinence, rather than a direct effect of alcohol policy change.^13^ Furthermore, there were significant sex differences in the cohort effects, reflecting the differences in social acceptability of alcohol consumption for males as compared to females.^13^

The gender norms affecting alcohol use are likely also relevant to tobacco consumption, as both alcohol consumption and tobacco consumption have historically varied strongly by sex in Finland, with prevalence being higher for males as compared to females for both substances.^6,13^ Indeed, additional research from Eastern Finland indicates that the cohort effects on smoking initiation did vary by sex, with prevalence steadily decreasing in males but increasing in females across birth years 1913–1957.^8^ Work in American samples found that the changes in tobacco use across birth cohorts differ for males and females, in that males in more recent birth cohorts smoke at lower rates than males in earlier cohorts, but the opposite is true when comparing females in different birth cohorts.^9^ Lastly, a study of tobacco initiation across Europe utilized age–period–cohort modeling, under the assumption that cohort effects were an additive combination of age and period effects.^11^ For Northern European females born in the 1950s, they found an excess of smoking initiation as compared to the rates expected under that assumption. In other words, they identified sex-specific cohort effects on smoking initiation.

As discussed, there is evidence that substance use and its underlying variability both depend on the degree to which use is encouraged or discouraged, be it through drug control policies or social norms.^9,10,13^ This suggests that if some policy is effective in limiting smoking behaviors, it also may change the composition of the population who does continue to smoke. This can be via “hardening” or “softening” of the population of smokers. The hardening hypothesis suggests that tobacco control policies are most effective in influencing smokers who are more willing or able to quit, leaving the remaining population of smokers made up of individuals less likely to quit.^14^ On the other hand, the softening hypothesis suggests that tobacco control policies result in a population of smokers with increasing willingness or ability to stop smoking.^15^ There is evidence that Finland may be experiencing a “softening” of smokers with increasingly recent birth cohorts,^15^ and there is broader evidence to suggest softening in other countries as well.^14,16–18^

We aimed to expand the existing literature and investigate cohort effects on tobacco consumption and the genetic and environmental influences underlying it in a Finnish sample. We utilized data from a Finnish twin cohort study, containing adult twins born between 1880 and 1957 and assessed up to four times. These research questions guided our investigation into cohort effects on tobacco consumption: (1) How has the prevalence of regular tobacco use and average consumption by users changed across age, time, and differed by sex? (2) Do we identify sex and/or cohort effects on the genetic and environmental influences on tobacco consumption in Finland? The project was preregistered on July 27, 2022, available at https://osf.io/s324t/.

## Methods

### Sample

We used data from the older Finnish Twin Cohort (*N* = 28 542), a population-based cohort study of twins born before 1958 and living in Finland at the time of inception. Individuals responded to questionnaires between one and four times, during the years 1975, 1981, 1990, and 2011.^19–21^ Demographic variables included age at assessment, sex, zygosity, and birth year. Participants of unknown zygosity were excluded; the final sample size included *N* = 26 116 individuals from 13 997 twin pairs. Birth cohorts were defined as 10-year bands (eg, 1901–1910, 1911–1920…) with the exception of those born prior to 1900, which were grouped as 1880–1900 due to small sample size. We also defined two larger birth cohorts assessed at similar ages (birth years 1901–1920 assessed in 1975 and birth years 1945–1957 assessed in 2011) to disentangle age effects from cohort effects.

### Measures

Cigarette smoking status was reported at each assessment via two items. The first item defined ever smoking; participants were asked if they had smoked more than 5–10 packs of cigarettes in their life. The second item defined regular smoking; participants were asked if they had ever smoked cigarettes regularly (daily or almost daily) regardless of whether they were smoking at the time of the survey or had quit. Based on available assessments for each individual, we created lifetime versions of these two variables to make our measure most similar to previous work^10^ for comparison purposes. If a participant, at any assessment, responded as yes to the ever-smoking or regular smoking item, they were coded as a lifetime regular cigarette user. This variable includes both current and former regular cigarette users.

Former and current regular cigarette smokers reported average daily consumption (either before quitting if former, or current) on an ordinal scale with response options as follows: none, less than 5, 5–9, 10–14, 15–19, 20–24, 25–39, and 40+ cigarettes per day. Individuals who reported never smoking, and individuals who reported ever but not regular smoking, were not asked the quantity item.

### Analyses

To evaluate how the patterns of cigarette consumption have changed over time, we utilized two approaches: a mixed-effects analysis and twin model variance decompositions. We used the R packages lme4,^22^ lmerTest,^23^ and MuMIn for mixed-effects models, the R packages GAMM^24^ and ggplot2^25^ for visualizations, and the R package OpenMx^26,27^ for twin model variance decompositions.

#### Research Question 1: Mixed-Effects Models

Mixed-effects analyses modeled quantity of tobacco consumption as a function of age utilizing all available longitudinal data, in which repeated measures were nested within individuals, who were then nested within twin pairs. We included linear and quadratic age effects, as substance use trajectories are nonlinear across the lifespan.^5,13^ We also included a main effect of sex, given historical differences in smoking rates between Finnish males and females.^6^ Lastly, we included a main effect of cohort and age × cohort and sex × cohort interaction effects.

#### Research Question 2: Twin Model Variance Decompositions

To evaluate cohort effects on the genetic and environmental influences underlying tobacco consumption, we utilized variance decompositions in a multigroup twin model using the direct symmetric model parameterization.^28^ Here, we compared males and females in two larger birth cohorts at one time point (birth years 1901–1920 assessed in 1975 and birth years 1945–1957 assessed in 2011, hereafter referred to as “earlier” and “later” cohorts, respectively) on lifetime regular use and quantity when smoking regularly.

Twin models leverage the genetic relatedness of monozygotic and dizygotic twins to partition variance of a trait into various underlying sources. Here, we considered additive genetic variation (A), shared environmental variation (C), and unique environmental variation (E) underlying initiation of regular smoking and quantity of cigarettes consumed when smoking regularly. To incorporate data from both lifetime regular use and quantity when smoking regularly, we used the causal–contingent–common (CCC) pathway models.^29–31^ The CCC is a multistage model in which the relationship between initiation (ie, regular smoking) and progression (ie, quantity of cigarettes) can be estimated by leveraging twin relatedness. Individuals who do not initiate a behavior are necessarily missing information on progression, and so the CCC compares progression among twins whose cotwins do and do not initiate.

The key parameters of interest are the degree to which genetic and environmental factors that influence initiation also influence progression (ie, the causal path), as well as the existence and magnitude of variation in progression of factors specific to progression. CCC models parameterize the causal pathway as an estimated coefficient that signifies the strength of relationship between initiation of regular smoking and quantity of cigarette consumption (see Figure S1). This coefficient is then multiplied by the magnitude of initiation variance components to generate the amount of variation in quantity due to initiation of regular smoking. The total variation in quantity is a combination of this shared variation and variation unique to quantity.

We proceeded with the full ACE multigroup CCC model in which the four sex × cohort groups were modeled simultaneously. All variance components were retained, regardless of whether the parameter’s confidence interval included 0, to avoid bias from model comparisons conducted in a reduced model. Both the mean of the latent liability distribution for initiation and the mean cigarette quantity were corrected for age at assessment. We then computed predicted thresholds at two ages (60 and 65) to facilitate comparisons of initiation likelihood. Comparative model fit was evaluated by log-likelihood ratio test, such that restricted models in which some estimated parameters were fixed to equivalence were compared to models in which the parameters are freely estimated. For all comparisons of interest, we first ran an omnibus test, restraining the parameter of interest to equality for all four sex × cohort groups. If the omnibus test were significant, we then ran further tests to identify the source of the effect (ie, sex differences and/or cohort differences).

### Sensitivity Analyses

We conducted non-preregistered sensitivity analyses to further examine age, period, and cohort (APC) effects,^11,32^ described in detail in Supplementary Methods.

## Results

### Descriptives

**Figure 1.**
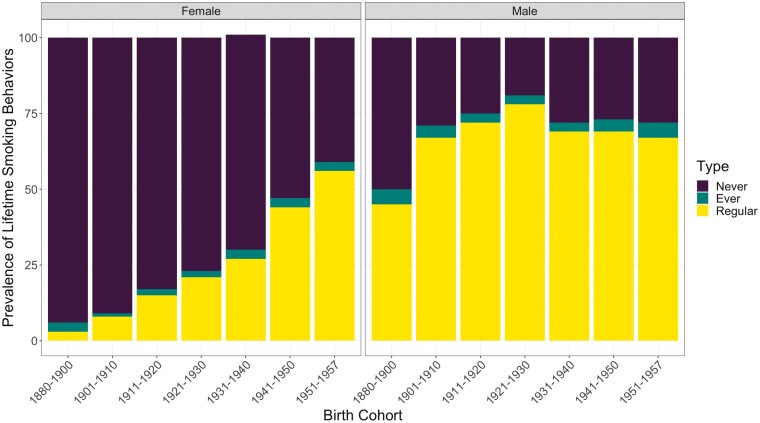
Percentages of lifetime ever and regular smoking plotted by birth cohort and sex. Never indicates individuals who have never smoked cigarettes, ever refers to individuals who have smoked but not transitioned to regular smoking, and regular refers to current and former regular cigarette smokers.

Lifetime prevalence of ever smoking and regular smoking were not stable over birth cohorts and sexes (see Figure 1). The figure suggests that males of all birth cohorts were more likely than females to have ever smoked or become regular smokers, and the prevalence of both ever and regular smoking increased in females over increasingly recent birth cohorts. Prevalence of lifetime ever and regular smoking was highest in males born between 1921 and 1930 and lowest among females born between 1880 and 1900. These trends suggested by Figure 1 are statistically evaluated in both the mixed-effects models and twin models.

Twin concordance in lifetime ever smoking and initiation of regular smoking, as well as twin correlations for quantity of cigarette consumption at each assessment year are presented in Table S1; these are presented stratified by zygosity and sex. Monozygotic twins tended to be more similar than dizygotic twins, indicating additive genetic influences on both traits.

For the subset of individuals used in CCC twin modeling (earlier cohort from birth years 1901–1920 assessed in 1975 and later cohort from birth years 1945–1957 assessed in 2011) descriptive statistics are presented in Table 1, split by sex and zygosity. The simple descriptive statistics suggest that smoking prevalence was highest in earlier cohort males and lowest in earlier cohort females, and that mean cigarette quantity was larger in males as compared to females. These differences were statistically evaluated in the twin models to best account for family structure.

**Table 1.** Descriptives for Earlier and Later Cohorts Used in CCC Model

	Earlier cohort in 1975				Later cohort in 2011			
Age mean (SD)	62.9 (5.3)				60.3 (3.8)			
Age range	55–75				53–67			
% Females	60%				51%			

	Earlier males		Earlier females		Later males		Later females	
Sample size	1054		1735		3501		4366	
Number of regular smokers (prevalence of regular smoking)	725 (70%)		194 (11%)		1980 (57%)		1789 (41%)	
Mean (SD) cigarette quantity	4.8 (1.5)		3.5 (1.3)		4.6 (1.5)		3.6 (1.4)	

	MZ	DZ	MZ	DZ	MZ	DZ	MZ	DZ
Twin tetrachoric correlation for regular smoking	0.54	0.45	0.82	0.43	0.80	0.53	0.74	0.54
Twin correlation for quantity [95% CI]	0.34 [0.20, 0.47]	0.28 [0.17, 0.39]	0.54 [0.44, 0.62]	0.21 [0.13, 0.29]	0.59 [0.52, 0.65]	0.36 [0.29, 0.42]	0.53 [0.47, 0.58]	0.36 [0.31, 0.41]

In twin correlations for quantity, nonsmoking twins are set to missing and the correlation is based on complete pairs only (ie, concordant smoking pairs). CCC = causal–contingent–common; CI = confidence interval; SD = standard deviation.

### Mixed-Effects Models

**Figure 2.**
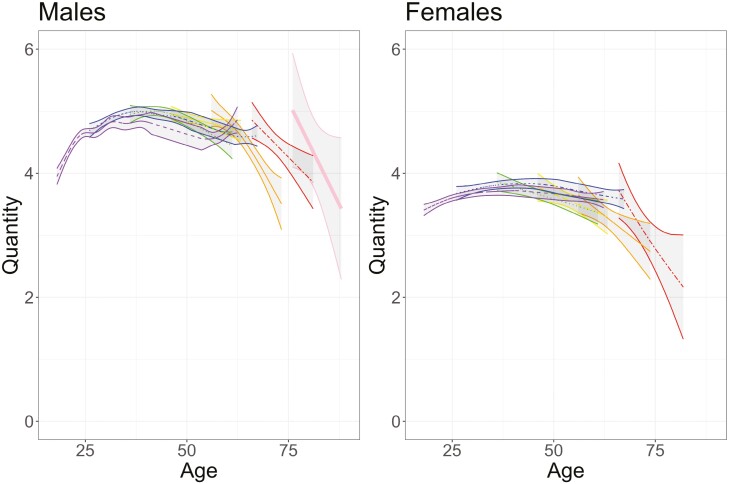
Ribbon plot generated from a generalized additive mixed model where tobacco consumption is regressed on age and smoothing parameters, separately by sex and cohort. Birth cohorts are defined as follows: pink/thick solid line = 1880–1900, red/two dash line = 1901–1910, orange/thin solid line = 1911–1920, yellow/long dash line = 1921–1930, green/dotted line = 1931–1940, blue/dot-dash line = 1941–1950, purple/dashed line = 1951–1957. The y-axis represents the ordinal quantity variable, with responses as follows: 1 = none, 2 = less than 5, 3 = 5–9, 4 = 10–14, 5 = 15–19, 6 = 20–24, 7 = 25–39, 8 = more than 40 cigarettes per day. Lines represent the predicted trajectory, and the ribbons represent the 95% confidence interval around the predicted trajectory.

Mixed-effects models evaluated the trajectory of cigarette consumption quantity with increasing age. We identified significant main effects of age, sex, and cohort, as well as significant interactions for all included terms. Table 2 contains details for all estimated parameters and Figure 2 provides a visualization of the predicted quantity by age, stratified by sex and birth cohort.

**Table 2.** Fixed-Effects Estimates From Mixed-Effects Model

	Estimate	SE	DF	*t*	*p*
Intercept	6.14	0.344	32 187	17.8	7.7 × 10^−71^
Cohort	−0.22	0.048	28 972	−4.7	2.6 × 10^−6^
Sex	−1.67	0.119	15 084	−14.0	4.2 × 10^−44^
Age	0.05	7.3 × 10^−3^	26 232	7.3	2.6 × 10^−13^
Age2	−7.6 × 10^−4^	4.5 × 10^−5^	21 266	−16.9	8.9 × 10^−64^
Education	−0.05	6.7 × 10^−3^	13 147	−7.9	2.2 × 10^−15^
Sex × cohort	0.09	0.020	14 769	4.8	1.8 × 10^−6^
Age × cohort	1.8 × 10^−3^	6.5 × 10^−4^	31 982	2.8	5.3 × 10^−3^

Models included random effects of individuals with repeated measures, nested within twin pairs. DF = degrees of freedom; SE = standard error.

Across all fixed effects included, the model explained 14% of the variation in cigarette quantity. Both linear (*b* = 0.05, *p* = 2.6 × 10^−13^) and quadratic (*b* = −7.6 × 10^−4^, *p* < 2 × 10^−16^) age effects were significant, confirming evidence of nonlinear changes in tobacco consumption across adulthood. Quantity increases with increasing age (positive linear age effect) but as individuals become increasingly older, the effect of age on quantity weakens (negative quadratic age effect) until the relationship between age and quantity eventually becomes negative. In Figure 2, this is indicated by the positive slopes at younger ages and negative slopes at later ages. We also identified a main effect of sex, indicating that males consumed higher quantities of cigarettes than females (*b* = −1.67, *p* < 2 × 10^−16^). The main effect of birth cohort indicated that quantity decreased with more recent birth cohorts (*b* = −0.22, *p* = 2.6 × 10^−6^). Lastly, there were significant age × cohort and sex × cohort interactions. Figure 2 provides a visualization of these interactions for interpretation. The sex × cohort interaction suggests that the effect of cohort (decreasing quantity with more recent birth cohorts) is weakened in females as compared to males. The age × cohort interaction indicates that the effect of age is weaker in earlier birth cohorts as compared to later birth cohorts.

### Twin Variance Decompositions

Full ACE CCC model results are presented in Table S2, and parameter comparisons are presented in Tables S3 (omnibus tests) and S4 (specific sex and cohort effects tests). We first tested sex and cohort differences in the predicted threshold for regular smoking at ages 60 and 65. There were no differences in predicted threshold between males and females in the earlier birth cohort (age 60 *p* = .68, age 65 *p* = .71), nor between earlier and later birth cohorts in females (age 60 *p* = .41, age 65 *p* = .42). On the other hand, thresholds differed significantly in males from earlier and later birth cohorts (age 60 *p* = .03, age 65 *p* = .04) and between males and females from the later birth cohort (age 60 *p* = 3.5 × 10^−3^, age 65 *p* = 3.0 × 10^−3^). These predicted thresholds were largest for later birth cohort males as compared to the other groups.

We also identified sex and cohort differences in predicted mean quantity at ages 60 and 65. Predicted mean quantity at 60 and 65 was larger in males than females for both the earlier (age 60 *p* = 3.3 × 10^−3^, age 65 *p* = 4.5 × 10^−3^) and later birth cohorts (age 60 *p* = 9.3 × 10^−19^, age 65 *p* = 3.8 × 10^−16^). Furthermore, earlier birth cohort males smoked more heavily than later birth cohort males (age 60 *p* = 1.5 × 10^−4^, age 65 *p* = .01), but there were no differences in predicted mean quantity between earlier and later birth cohorts in females (age 60 *p* = .06, age 65 *p* = .06).

We identified significant contributions from initiation to variance in quantity for earlier cohort females, later cohort males, and later cohort females, see Table S2 for CCC model estimates. Whereas for earlier cohort males, the confidence interval was very wide and included both 0, suggesting the variation in progression may be independent from the variation in initiation, and was close to 1 suggesting a single liability for initiation and quantity in earlier males. The magnitude of the causal path did not significantly differ between all four groups (*p* = .22), despite the substantially smaller point estimate in the earlier cohort males, possibly due to the large standard error around it. The variance decomposition for regular smoking similarly did not differ between all four sex × cohort groups (ie, simultaneously constraining A, C, and E between all four groups, *p* = .15), though confidence intervals for the earlier cohort estimates were quite wide and some included 0.

Beyond the variance in quantity shared with variance in regular smoking, we identified variation unique to quantity. Specific variation in quantity was largely due to unique environmental and additive genetic sources (all groups around *h*^2^ ~ 0.4 and *e*^2^ ~ 0.6; see Table S2 for exact estimates and confidence intervals) with minimal contributions from the shared environment. The decomposition of variation specific to smoking quantity did not differ between sex × cohort groups (see Table S3 for comparisons).

The amount of variation specific to quantity was larger in males as compared to females in the later birth cohort (*p* = 1.1 × 10^−4^) as well as in the earlier birth cohort (*p* = 4.9 × 10^−3^). Conversely, the amount of variation specific to quantity did not differ between earlier and later cohorts in both males (*p* = .79), and females (*p* = .21). In other words, we identified sex differences, but not cohort effects, in the amount of variation specific to quantity. The total variation in quantity (variance specific to quantity plus variation in quantity shared with initiation) was largest in later birth cohort males, as compared to both later birth cohort females (*p* = .04) as well as earlier birth cohort males (*p* = 6.1 × 10^−4^); therefore, we identified both sex and cohort effects in the total variation underlying quantity.

In summary, we detected significant sex and cohort effects in the amounts of variation underlying quantity as well as sex and cohort effects in the threshold of the liability to initiate regular smoking and its relationship with age, as well as predicted mean cigarette quantity. We did not identify significant sex or cohort effects in the sources of variation underlying either initiation of regular smoking or cigarette quantity.

### Sensitivity Analyses

Sensitivity analyses are described in detail in the Supplementary Results. Briefly, for cigarette quantity, cohort effects can be expressed as interaction of age and period effects but this is not the case for initiation of regular smoking (Supplementary Figures S2 and S3, Supplementary Tables S5 and S6).

## Discussion

We used longitudinal mixed-effects models and causal–common–contingent twin models to evaluate sex and cohort effects on tobacco consumption and the variation underlying it. Both the mixed-effects and twin variance decomposition models produced evidence that the age and sex effects on tobacco consumption vary by birth cohort. We found that across more recent birth cohorts smoking prevalence increased in females, though smoking prevalence in males was consistently higher than that of females. We identified both linear and quadratic effects of age; cigarette quantity increased with age, but since the quadratic effect was negative, the rate at which cigarette quantity increases with age becomes smaller over time, eventually turning negative. Additionally, we found that the positive relationship between age and quantity of tobacco consumption became stronger as birth cohorts became more recent, as indicated by the significant positive age × cohort interaction in the mixed-effects model.

More focused twin analyses on the subset of participants born between 1901 and 1920 and assessed in 1975 and participants born between 1945 and 1957 and assessed in 2011 (earlier and later cohorts) also revealed significant sex and cohort effects on tobacco consumption, specifically we identified differences in the liability to initiate regular smoking and mean smoking quantity between the sex × cohort groups. The relationship between age and latent liability to initiate regular smoking was different for later birth cohort males as compared to the other three sex × cohort groups, as were the intercept of the regression of age on latent liability to initiate regular smoking and the predicted thresholds at ages 60 and 65. This result is consistent with changes in smoking prevalence by birth cohort differing by sex.

Additionally, we identified sex and cohort effects on the variation underlying tobacco consumption. The amount of variation in cigarette quantity differed between cohorts and sexes. Earlier and later males had greater amounts of variation specific to quantity as compared to earlier and later females, respectively. This represents a sex effect, but no cohort effect on variation specific to cigarette quantity; males have greater variation than females across both birth cohorts.

Furthermore, the total variation in cigarette quantity (variance specific to quantity plus variation in quantity shared with initiation) was largest in later birth cohort males, as compared to both later birth cohort females and earlier birth cohort males. Therefore, we identified both sex and cohort effects in that the later cohort males were distinct from other groups. However, despite this difference in amounts of variation, the heritability and environmental influences on cigarette quantity did not differ between groups, nor did the variance decomposition for regular smoking differ between groups.

This work extends existing research on Finnish smoking behaviors at the population level,^6^ research on alcohol cohort effects in a genetically informative Finnish sample,^13^ and tobacco cohort effects research in a genetically informative American sample.^9,10^ Our estimates of heritability and environmental effects for both initiation of regular smoking and cigarette quantity are in line with previously published meta-analytic estimates^33^ and estimates from large studies, including those in Finland.^34,35^

Various other studies have found changes in smoking prevalence in more recent birth cohorts.^10,36,37^ In line with broader population-level research in Finland specifically,^6,38^ we found that across more recent birth cohorts smoking prevalence increased in females, though smoking prevalence and cigarette quantity in males were consistently higher than that of females. The interaction effects are potentially the most interesting component of the mixed-effects model of cigarette quantity; we identified both age × cohort and sex × cohort interactions.

The sex × cohort interaction suggests, as compared to males, cigarette quantity decreases at a slower rate in females with increasingly recent birth years. The age × cohort interaction indicates that the rate at which cigarette quantity increases with age accelerates as birth year becomes more recent. Additionally, as there are both significant positive linear and negative quadratic age effects, cigarette quantity first increases with age, then decreases. The significant age × cohort interaction then implies that the inflection age where quantity changes from increasing to decreasing would vary by birth cohort, where it would be younger for earlier birth cohorts and older for later birth years. Analyses in the same sample have identified similar age × cohort and sex effects on alcohol consumption; therefore, it is possible that some changes in social norms may influence both the use of alcohol and tobacco.^13^

Our results do not support the existence of sex and cohort effects on the heritability of regular smoking initiation, though the estimates were accompanied by wide confidence intervals, suggesting a possible lack of power. There is mixed evidence in the existing literature for the existence of sex and cohort effects on heritability.^9,10,12,39^ These differences in results are not necessarily inconsistent with each other; rather, heritability is a population-level statistic that applies to a specific group at a specific time, where time can represent age, period, and/or cohort. The cited studies represent a range of assessment ages, birth cohorts, phenotype definitions, and locations and these differences in study design may explain differences in whether sex and cohort effects were or were not identified. Furthermore, the studies vary in terms of which stage of the tobacco epidemic the study population was in at the time.^40^ The present study, for example, approximately spans WHO stages II–IV and Finland is a forerunner in tobacco control efforts.^41–44^ In this context, the timing and type of changes in the tobacco environment (policy, social norms, etc.) relative to the age and timing of assessment are likely also very relevant to whether or not cohort effects are identified.

We also did not identify significant differences in the relationship between initiation of regular smoking and quantity of cigarette consumption between sex × cohort groups, but we note again the potential for power issues in this comparison. This result though is consistent with other studies suggesting strong overlap between initiation and progression of tobacco behaviors.^45,46^

Lastly, we conducted sensitivity analyses to disentangle APC effects. Our sensitivity analyses of cigarette quantity suggest that cohort effects can be represented as the interaction of age and period effects. Our sensitivity analyses of initiation of regular smoking indicate the opposite, that there are cohort effects acting on initiation of regular smoking above and beyond the interaction of age and period effects. It is plausible that environmental factors relevant to tobacco use act as a period effect on quantity of cigarettes used, but a cohort effect on initiation of regular smoking. For example, if an environmental factor like antismoking social norms works to discourage smoking, it could act by reducing rates of initiation in individuals in the specific age range likely to initiate, as well as reduce quantity across individuals of all ages who already smoke.

### Limitations

Our results indicated a possible combination of age, period, and/or cohort effects on the prevalence and quantity of smoking and its underlying variation. Policy changes represent a source of environmental variation that could lead to differences between cohorts, but neither of these analyses can conclusively determine whether policy change caused the observed differences, as compared to broader social changes. Helakorpi et al. had data on even more recent birth years than we presented here and were therefore more able to demonstrate potentially causal links between the decrease in smoking prevalence and the enactment of the 1976 Tobacco Control Act.^6^ Our results are consistent with theirs, but all individuals in our study were beyond the average age of tobacco initiation by the time this stricter tobacco legislation was in place. On the other hand, Helakorpi et al. did not include genetically informative data, such as families or twin pairs, and thus only changes in prevalence could be linked to tobacco policy, not any changes in its underlying variation.^6^

Additionally, as our first assessment was in 1975 and the earliest birth years were 1880–1900, there may be issues with loss through mortality. It is likely that our analyses underestimated lifetime prevalence of regular tobacco smoking and average cigarette consumption in the earliest birth years, as the heaviest smokers were more likely to die from smoking-related causes prior to the first assessment.

## Conclusions

The prevalence of regular smoking and quantity of cigarette consumption have varied with respect to age and sex across birth cohorts. Prevalence of smoking increased substantially in females and cohort differences in the quantity of tobacco smoked were more pronounced in males than in females. Lastly, there was greater variation contributing specifically to smoking quantity in earlier and later birth cohort males as compared to females, as well as larger total variation in cigarette quantity in males in later birth cohorts as compared to all other groups. Future genetically informative work is needed in later birth cohorts to evaluate how large-scale tobacco policies may influence tobacco consumption and its underlying variation.

## Supplementary Material

Supplementary material is available at *Nicotine and Tobacco Research* online.

ntae091_suppl_Supplementary_Figures_1_Tables_1-3

## Data Availability

Data are not publicly available due to data security regulations by the University of Helsinki. Code for the two primary models (mixed-effects model, base CCC model) are available at https://osf.io/s324t/.
