# Supplementary material for: Cohort Effects on Tobacco Consumption and Its Genetic and Environmental Variance Among Finnish Adults Born Between 1880 and 1957
Source: Nicotine Tob Res. 2024 Apr 17;26(12):1607–15. doi: 10.1093/ntr/ntae091 (PMC11581999; doi:10.1093/ntr/ntae091)
Supplement: ntae091_suppl_Supplementary_Figures_1_Tables_1-3 [file ntae091_suppl_supplementary_figures_1_tables_1-3.docx]

Supplemental Material

Cohort Effects on Tobacco Consumption and its Genetic and Environmental Variance Among Finnish Adults born between 1880-1957

Stephanie Zellers, PhD^1^

Hermine H.M. Maes, PhD^2^

Antti Latvala, PhD^3^

Jaakko Kaprio, MD PhD^1^

1. Institute for Molecular Medicine Finland, University of Helsinki

2. Virginia Institute for Psychiatric and Behavioral Genetics, Department of Human and Molecular Genetics, Psychiatry and Massey Cancer Center, Virginia Commonwealth University

3. Institute of Criminology and Legal Policy, University of Helsinki

**Author Note**

Stephanie Zellers <https://orcid.org/0000-0001-8927-3483>

We have no known conflict of interest to disclose.

Correspondence concerning this article should be addressed to Stephanie Zellers, Institute for Molecular Medicine Finland, University of Helsinki, P.O. Box 20 Helsinki, Finland 00014 Contact: [stephanie.zellers@helsinki.fi](mailto:stephanie.zellers@helsinki.fi)

**SUPPLEMENTAL METHODS**

We conducted non-preregistered sensitivity analyses to further examine age, period, and cohort (APC) effects. Age, period, and cohort are always perfectly correlated and therefore there is an identification problem that cannot be solved without making theory-based assumptions about one of the effects in APC models. Tobacco policy changes may be one example of something that could be represented as a period effect or cohort effect and it is difficult to determine which with our data.

As a sensitivity test for Research Question 1, we reran the mixed effects model predicting cigarette quantity with main effects of age and period (assessment year) and utilized the epidemiological assumption that cohort effects are the interaction of age and period effects^1^. We then examined the model residuals by birth year to evaluate whether that assumption held^2^.

As a second sensitivity test for Research Question 2, we ran a mixed effects model predicting initiation of regular smoking with main effects of age and period (assessment year) in the same subsample of individuals used in the twin analysis. We again utilized the definition of cohort effects as the interaction of age and period and examined the model residuals by birth year to evaluate whether that assumption held.

**SUPPLEMENTAL RESULTS**

We conducted sensitivity analyses of both the mixed effects model and twin models to test if cohort effects could be expressed as the interaction of age and period. In the mixed effects model, the sensitivity analysis was quite similar in methodology to the original analysis. Full results are presented in Supplemental Table 5 and Supplemental Figure 2. Both age and period significantly predicted cigarette quantity, as did their interaction (cohort effect). When examining the model residuals, residuals increased in absolute size with increasing birth year, but this occurred symmetrically. In other words, there was no bias in the residuals across birth years. This suggests that the assumption holds, or in other words, that for cigarette quantity, cohort effects are the interaction of age and period effects.

On the other hand, the sensitivity mixed-effects analysis for the twin model was less analogous to the primary analysis. Full results are presented in Supplemental Table 6 and Supplemental Figure 3. Age significantly predicted initiation of regular smoking, but period and the interaction term did not. When examining the model residuals, the scatterplot was not symmetric and birth year significantly predicted the residuals. This indicates that for initiation of regular smoking, cohort effects cannot be expressed as the interaction of age and period effects. In other words, it is plausible that there are cohort effects acting on regular cigarette initiation, unique from age and period effects.


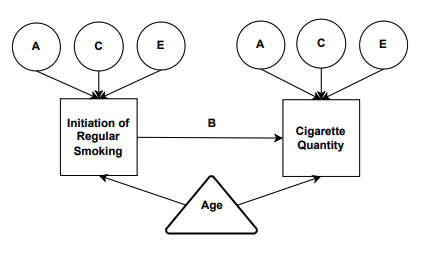


*Supplemental Figure 1.* Representative path diagram of CCC model, here B represents the causal path from initiation to progression. A, C, and E represent the sources of variation (additive genetic, shared environment, and unique environment) and age signifies an effect of age as a covariate on the mean. This model was estimated separately for each cohort and sex combination (four-group model).


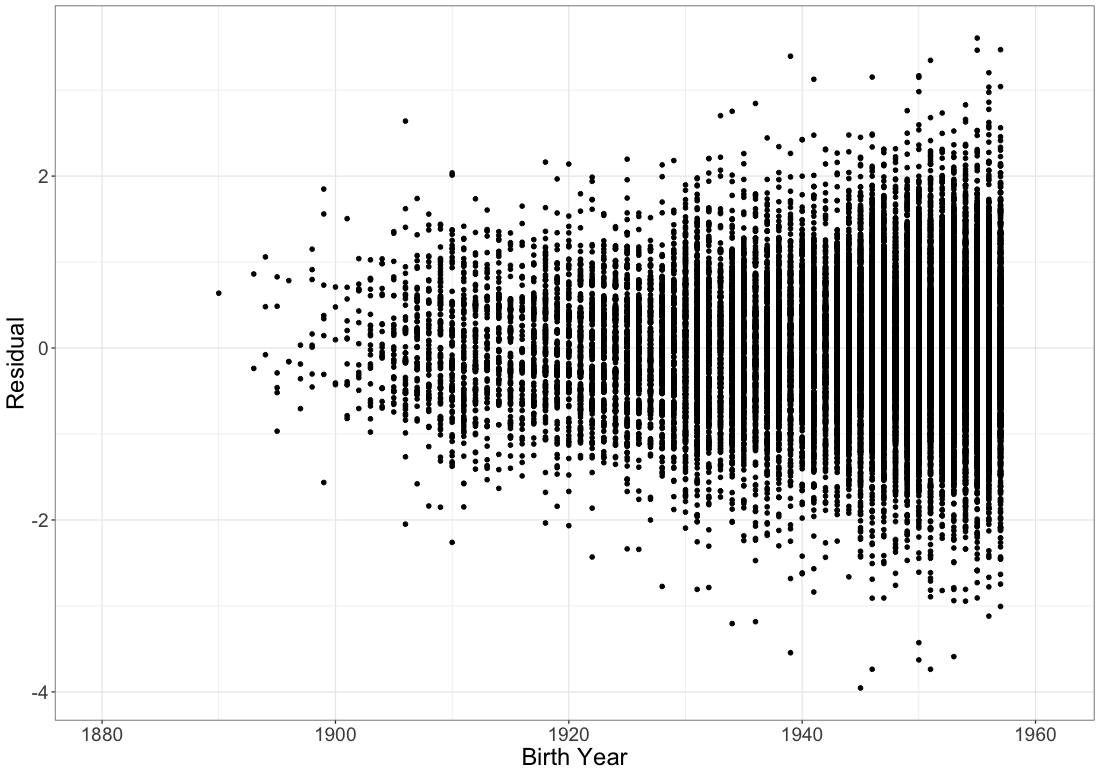


*Supplemental Figure 2.* Scatterplot of residuals by birth year from sensitivity analysis of cigarette quantity and age, period, cohort effects. Birth year was not significantly related to residuals.


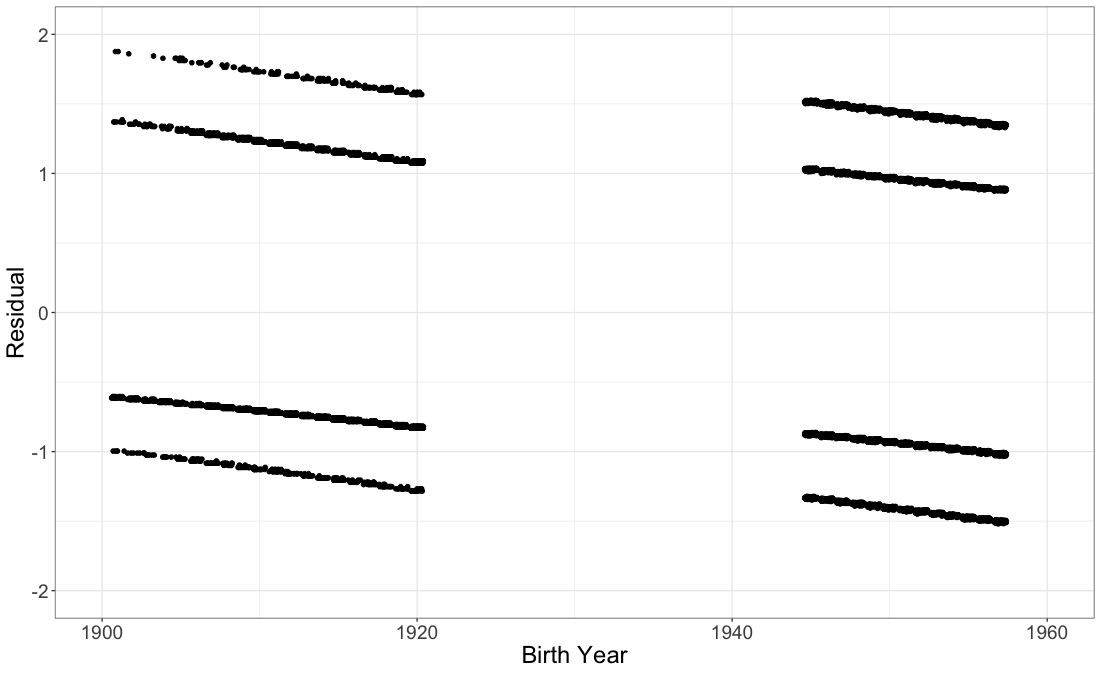


*Supplemental Figure 3.* Scatterplot of residuals by birth year from sensitivity analysis of initiation of regular smoking and age, period, cohort effects. Birth year significantly predicted residuals.

| Supplemental Table 1: Full Sample Twin Correlations Split by Sex and Zygosity | | | | |
| --- | --- | --- | --- | --- |
|  | Male MZ | Male DZ | Female MZ | Female DZ |
| Lifetime Ever Smoking Tetrachoric Correlation | 0.83 | 0.54 | 0.86 | 0.68 |
| Lifetime Regular Smoking Tetrachoric Correlation | 0.82 | 0.54 | 0.85 | 0.69 |
| 1975 Tobacco Quantity Correlation [95% CI] | 0.48  [0.42, 0.53] | 0.28  [0.24, 0.32] | 0.54  [0.47, 0.60] | 0.32  [0.26, 0.38] |
| 1981 Tobacco Quantity Correlation [95% CI] | 0.45  [0.39, 0.51] | 0.27  [0.23, 0.32] | 0.49  [0.42, 0.55] | 0.27  [0.20. 0.33] |
| 1990 Tobacco Quantity Correlation [95% CI] | 0.36  [0.26, 0.45] | 0.21  [0.14, 0.29] | 0.49  [0.39, 0.59] | 0.30  [0.21, 0.39] |
| 2011 Tobacco Quantity Correlation [95% CI] | 0.36  [0.23, 0.48] | 0.20  [0.08, 0.30] | 0.46  [0.34, 0.57] | 0.25  [0.13, 0.35] |

| Supplemental Table 2: Parameter Estimates from Full ACE CCC Twin Model | | | | |
| --- | --- | --- | --- | --- |
| Parameter | Estimate [95% CI] | | | |
|  | Older Male | Older Women | Younger Male | Younger Women |
| A Regular Smoking (Standardized) | 0.21  [-0.35, 0.72] | 0.83  [0.37, 1.27] | 0.54  [0.30, 0.77] | 0.51  [0.31, 0.71] |
| C Regular Smoking (Standardized) | 0.34  [-0.07, 0.72] | -0.01  [-0.39, 0.35] | 0.25  [0.05, 0.44] | 0.25  [0.08, 0.41] |
| E Regular Smoking (Standardized) | 0.46  [-.28, 0.69] | 0.17  [0.07, 0.34] | 0.21  [0.14, 0.30] | 0.24  [0.18, 0.31] |
| Threshold  Age Beta | -0.02  [-0.04, -0.01] | -0.03  [-0.05, -0.02] | 0  [-0.02, 0.02] | -0.04  [-0.05, -0.02] |
| Predicted Threshold at 60 | -3.52  [-5.96, -1.09] | -2.83  [-5.10, -0.60] | -0.10  [-2.11, 1.90] | -4.00  [-5.63, -2.37] |
| Predicted Threshold at 65 | -3.64  [-6.19, -1.11] | -2.99  [-5.36, -0.66] | -0.10  [-2.20, 1.99] | -4.18  [-5.88, -2.48] |
| Causal  Path | 0.29  [-0.40, 0.94] | 1*  [0.42, 1*] | 0.84  [0.57, 1*] | 0.93  [0.67, 1*] |
| Total Variance in Quantity | 2.09  [1.84, 2.60] | 2.22  [1.56, 2.59] | 2.77  [2.46, 3.12] | 2.33  [2.03, 2.57] |
| Total A Quantity (Standardized) | 0.36  [-0.12, 0.81] | 0.57  [-0.36, 1.29] | 0.39  [0.09, 0.66] | 0.48  [0.24, 0.71] |
| Total C Quantity (Standardized) | 0.05  [-0.32, 0.39] | 0.01  [-0.58, 0.61] | 0.06  [-0.16, 0.28] | 0.09  [-0.11, 0.27] |
| Total E Quantity (Standardized) | 0.59  [0.44, 0.79] | 0.41  [0.21, 0.82] | 0.55  [0.45, 0.67] | 0.43  [0.35, 0.53] |
| Specific Variance in Quantity | 2.01  [1.61, 2.30] | 1.22  [0.94, 1.59] | 2.06  [1.83, 2.30] | 1.47  [1.30, 1.68] |
| Specific A Quantity (Standardized) | 0.37  [-0.13, 0.84] | 0.36  [-1.20, 1.62] | 0.33  [-0.06, 0.71] | 0.46  [0.08, 0.83] |
| Specific C Quantity (Standardized) | 0.03  [-0.35, 0.40] | 0.02  [-1.01, 1.09] | 0  [-0.30, 0.29] | -0.01  [-0.31, 0.28] |
| Specific E Quantity (Standardized) | 0.60  [0.44, 0.80] | 0.62  [0.25, 1.34] | 0.67  [0.54, 0.82] | 0.55  [0.43, 0.69] |
| Quantity  Age Beta | -0.03  [-0.06, -0.01] | -0.04  [-0.09, -0.01] | 0.01  [-0.02, 0.03] | -0.03  [-0.05, 0] |
| Predicted Quantity Mean at 60 | 4.69  [4.35, 5.04] | 1.86  [1.65, 2.83] | 3.96  [3.77, 4.16] | 2.73  [2.58, 2.96] |
| Predicted Quantity Mean at 65 | 4.54  [4.16, 4.92] | 1.65  [1.39, 2.68] | 3.99  [3.76, 4.22] | 2.59  [2.38, 2.88] |

Note: * indicates an upper or lower bound on a free parameter. Upper bounds of standardized parameters may exceed 1 as an artefact of the direct symmetric model parameterization.

| Supplemental Table 3: Omnibus Tests of Sex and Cohort Differences in CCC Model | | | |
| --- | --- | --- | --- |
| Parameter Compared | **Difference in Log-Likelihood** | **df** | **p-value** |
| ACE Decomposition Regular Smoking | 9.38 | 6 | 0.15 |
| Threshold  Age Beta | **11.46** | **3** | **0.0095** |
| Predicted Threshold at 60 | **9.36** | **3** | **0.02** |
| Predicted Threshold at 65 | **9.41** | **3** | **0.02** |
| Causal  Path | 4.40 | 3 | 0.22 |
| Total Variance in Quantity | **8.49** | **3** | **0.04** |
| Total A Quantity (Standardized) | 0.46 | 3 | 0.93 |
| Total C Quantity (Standardized) | 0.08 | 3 | 0.99 |
| Total E Quantity (Standardized) | 6.57 | 3 | 0.09 |
| Specific Variance in Quantity | **23.62** | **3** | **3.0×10^-5^** |
| Specific A Quantity (Standardized) | 0.24 | 3 | 0.97 |
| Specific C Quantity (Standardized) | 0.04 | 3 | 0.99 |
| Specific E Quantity (Standardized) | 1.47 | 3 | 0.69 |
| Quantity  Age Beta | 5.68 | 3 | 0.13 |
| Predicted Quantity Mean at 60 | **154.52** | **3** | **2.8×10^-33^** |
| Predicted Quantity Mean at 65 | **113.19** | **3** | **2.3×10^-24^** |

Note: Omnibus tests presented for all parameters. A significant p-value indicates that model fit worsens when equating groups and therefore there are some sex and/or cohort effects on that parameter; bolded p-values are significant, interpreted in the main manuscript, and followed up with more specific tests.

| Supplemental Table 4: Tests of Sex and Cohort Effects following Significant Omnibus Tests | | | | | | | | |
| --- | --- | --- | --- | --- | --- | --- | --- | --- |
|  | **Tests for Sex Differences** | | | | **Tests for Cohort Differences** | | | |
|  | **Groups**  **Equated** | **Difference in Log-Likelihood** | **df** | **p-value** | **Groups**  **Equated** | **Difference in Log-Likelihood** | **df** | **p-value** |
| Threshold  Age Beta | Earlier | 0.37 | 1 | 0.54 | Males | 3.50 | 1 | 0.06 |
|  | **Later** | **10.63** | **1** | **1.1×10^-3^** | Females | 0.05 | 1 | 0.83 |
| Predicted Threshold at 60 | Earlier | 0.17 | 1 | 0.68 | **Males** | **4.49** | **1** | **0.03** |
|  | **Later** | **8.52** | **1** | **3.5×10^-3^** | Females | 0.68 | 1 | 0.41 |
| Predicted Threshold at 65 | Earlier | 0.14 | 1 | 0.71 | **Males** | **4.44** | **1** | **0.04** |
|  | **Later** | **8.84** | **1** | **3.0×10^-3^** | Females | 0.64 | 1 | 0.42 |
| Total Variance in Quantity | Earlier | 0.27 | 1 | 0.61 | **Males** | **11.74** | **1** | **6.1×10^-4^** |
|  | **Later** | **4.05** | **1** | **0.04** | Females | 0.24 | 1 | 0.62 |
| Specific Variance in Quantity | **Earlier** | **7.91** | **1** | **4.9×10^-3^** | Males | 0.07 | 1 | 0.79 |
|  | **Later** | **14.95** | **1** | **1.1×10^-4^** | Females | 1.58 | 1 | 0.21 |
| Predicted Quantity Mean at 60 | **Earlier** | **8.66** | **1** | **3.3×10^-3^** | **Males** | **14.35** | **1** | **1.5×10^-4^** |
|  | **Later** | **78.21** | **1** | **9.3×10^-19^** | Females | 3.58 | 1 | 0.06 |
| Predicted Quantity Mean at 65 | **Earlier** | **8.06** | **1** | **4.5×10^-3^** | **Males** | **6.04** | **1** | **0.01** |
|  | **Later** | **66.35** | **1** | **3.8×10^-16^** | Females | 3.58 | 1 | 0.06 |

Note: Sex and cohort differences presented for the parameters for which the omnibus test was significant. A significant p-value indicates that model fit worsens when equating groups and therefore that parameter is significantly different between those groups; bolded p-values are significant and interpreted in the main manuscript. With respect to groups constrained, “males” refers to a parameter constraint between earlier and later birth cohort males; “females” refers to a parameter constraint between earlier and later birth cohort females; “earlier” refers to a parameter constraint between earlier cohort males and females; “later” refers to a parameter constraint between later cohort males and females.

| Supplemental Table 5: Fixed Effects Estimates from Cigarette Quantity Sensitivity Analysis | | | | | |
| --- | --- | --- | --- | --- | --- |
|  | **Estimate** | **SE** | **DF** | **t** | **p** |
| Intercept | 4.52 | 0.08 | 29602 | 56.3 | < 5×10^-300^ |
| Period | -0.09 | 0.03 | 31150 | -3.0 | 2.5×10^-3^ |
| Sex | -1.10 | 0.02 | 13329 | -46.9 | < 5×10^-300^ |
| Age | 0.08 | 3.8×10^-3^ | 31841 | 21.1 | 2.5×10^-98^ |
| Age^2^ | -9.4×10^-4^ | 5.0×10^-5^ | 30605 | -18.7 | 1.1×10^-77^ |
| Education | -0.05 | 6.7×10^-3^ | 13247 | -7.4 | 1.1×10^-13^ |
| Age×Period (Cohort) | 1.2×10^-3^ | 6.1×10^-4^ | 32284 | 2.0 | 0.04 |

Note: Models included random effects of individuals with repeated measures,

nested within twin pairs.

| Supplemental Table 6: Fixed Effects Estimates from Regular Cigarette Initiation Sensitivity Analyses | | | | |
| --- | --- | --- | --- | --- |
|  | **Estimate** | **SE** | **z** | **p** |
| Intercept | 3.15 | 1.09 | 2.89 | 3.8×10-3 |
| Sex | -1.13 | 0.04 | -27.23 | 2.7×10^-163^ |
| Age | -0.04 | 0.02 | -2.35 | 0.02 |
| Period | 0.22 | 0.64 | 0.34 | 0.73 |
| Age×Period (Cohort) | 4.8×10^-3^ | 0.01 | 0.46 | 0.64 |

Note: Models included random effects of individuals with repeated measures,

nested within twin pairs.

**Supplemental References**

1. Keyes KM, Utz RL, Robinson W, Li G. What is a cohort effect? Comparison of three statistical methods for modeling cohort effects in obesity prevalence in the United States, 1971–2006. *Soc Sci Med*. 2010;70(7):1100-1108. doi:10.1016/j.socscimed.2009.12.018

2. Marcon A, Pesce G, Calciano L, et al. Trends in smoking initiation in Europe over 40 years: A retrospective cohort study. *PLOS ONE*. 2018;13(8):e0201881. doi:10.1371/journal.pone.0201881
